# Drug-related deaths among housed and homeless individuals in the UK and the USA: comparative retrospective cohort study

**DOI:** 10.1192/bjp.2023.111

**Published:** 2023-12

**Authors:** Emmert Roberts, Caroline Copeland, Keith Humphreys, Chelsea L. Shover

**Affiliations:** National Addiction Centre, Institute of Psychiatry, Psychology & Neuroscience, King's College London, London, UK; and Department of Psychiatry and Behavioral Sciences, Stanford University School of Medicine, Stanford, California, USA; Institute of Pharmaceutical Science, King's College London, London, UK; Veterans Affairs Palo Alto Health Care System, Palo Alto, California, USA; and Department of Psychiatry and Behavioral Sciences, Stanford University School of Medicine, Stanford, California, USA; David Geffen School of Medicine, Division of General Internal Medicine and Health Services Research, University of California – Los Angeles (UCLA), Los Angeles, California, USA

**Keywords:** Drug-related deaths, homelessness, inequity, mortality, overdose

## Abstract

**Background:**

The UK and USA currently report their highest number of drug-related deaths since records began, with higher rates among individuals experiencing homelessness.

**Aims:**

Given that overdose prevention in homeless populations may require unique strategies, we evaluated whether substances implicated in death differed between (a) housed decedents and those experiencing homelessness and (b) between US and UK homeless populations.

**Method:**

We conducted an internationally comparative retrospective cohort study utilising multilevel multinomial regression modelling of coronial/medical examiner-verified drug-related deaths from 1 January 2012 to 31 December 2021. UK data were available for England, Wales and Northern Ireland; US data were collated from eight county jurisdictions. Data were available on decedent age, sex, ethnicity, housing status and substances implicated in death.

**Results:**

Homeless individuals accounted for 16.3% of US decedents versus 3.4% in the UK. Opioids were implicated in 66.3 and 50.4% of all studied drug-related deaths in the UK and the USA respectively. UK homeless decedents had a significantly increased risk of having only opioids implicated in death compared with only non-opioids implicated (relative risk ratio RRR = 1.87, 95% CI 1.76–1.98, *P* < 0.001); conversely, US homeless decedents had a significantly decreased risk (RRR = 0.37, 95% CI 0.29–0.48, *P* < 0.001). Methamphetamine was implicated in two-thirds (66.7%) of deaths among US homeless decedents compared with 0.4% in the UK.

**Conclusions:**

Both the rate and type of drug-related deaths differ significantly between homeless and housed populations in the UK and USA. The two countries also differ in drugs implicated in death. Targeted programmes for country-specific implicated drug profiles appear warranted.

In 2021 both the UK and the USA reported their highest number of drug-related deaths since records began.^1,2^ Research from each country has consistently demonstrated disproportionate drug-related harms in vulnerable and marginalised groups, particularly individuals experiencing homelessness.^3^

Homeless populations can often experience multiple disadvantage, including substantial barriers to accessing mental and behavioural health support.^4^ They may also form communities or subcultures with their own norms around drugs and drug use.^5^ Due in part to these phenomena, unique drug consumption patterns may develop that disproportionately affect the likelihood of drug-related death, with previously observed trends including increased consumption of stimulants to remain vigilant and prevent theft of belongings.^6,7^ Developing an understanding of differential fatal drug use between housed individuals and those experiencing homelessness could assist in understanding drug-related harm trajectories and aid in targeting preventive interventions to people with specific drug-use profiles. This may ultimately help reduce the substantial health inequities faced by this highly vulnerable and stigmatised group.^3^ Despite widespread national reporting of publicly available data relating to drug-related deaths, few jurisdictions routinely collect or report harms stratified by housing or homelessness status, with previous research often limited to small local samples or subpopulations with particular health conditions.^1,8,9^ Reports also often rely on broad pharmacological classes of substances implicated in death, preventing a clear understanding of actual drug use patterns or which specific substances, or groups of substances, are deemed causative of death.^10,11^ Consequently, a complete understanding of how deaths associated with drug use are differentially experienced within the homeless population is currently lacking in both the UK and the USA. Given the concerning recent escalation in drug-related death rates in both countries, and their disproportionate concentration within this population,^3^ a more granular understanding of the specific substances and characteristics associated with death is warranted.

To address this gap, we aimed to conduct an internationally comparative retrospective cohort study utilising coronial/medical examiner records to examine the population of people dying due to drug-related causes in both the UK and the USA. This aimed to determine any differences in sociodemographic characteristics and substances implicated in death between individuals who are housed and those experiencing homelessness at the time of their death within samples in the USA and the UK and any differences between each country's homeless populations. We hypothesised that, when compared with housed individuals, those experiencing homelessness would be on average younger, have higher proportions identifying as male and have higher proportions of people with the following substance use profiles implicated in death: (a) only non-opioid substances implicated, (b) only opioids implicated and (c) any opioid plus any non-opioid substance implicated. Additionally, we hypothesised there would be significant differences in drug-use and sociodemographic profiles between people experiencing homelessness in the USA compared with the UK. We assume this based on broader differences in North American versus European drug markets and the lower level of social welfare provision in the USA compared with the UK.

## Method

This study is reported according to the Strengthening the Reporting of Observational Studies in Epidemiology (STROBE) statement^12^ (the completed checklist is shown in Supplementary Table 1, available at https://dx.doi.org/10.1192/bjp.2023.111). The study protocol and statistical analysis plan were pre-registered on the Open Science Framework (OSF) and are available at https://osf.io/43ty5.

We conducted a comparative retrospective cohort study using records drawn from two data-sets. The US data were compiled by researchers at the University of California – Los Angeles (UCLA) from voluntary submission of coronial/medical examiner records from 30 jurisdictions across the USA, a sample that includes approximately 20% of the US population and has been previously described.^13^ Only jurisdictions that collect and report housing status were included in this analysis (*n* = 8, approximately 6% of the US population).^14^ Of note, the national source for overdose mortality data in the USA, the Centers for Disease Control and Prevention's Wide-Ranging Online Database for Epidemiologic Research (CDC WONDER), does not include housing status; it is therefore not possible to conduct a national analysis.^8^ The time frame of deaths recorded varied between jurisdictions but ranged from 1 January 2012 to 31 December 2021 (Table 1). Deaths were included in the US data-set if the death was verified as drug-related by the coroner/medical examiner or if, following case review, the stated death certificate ‘cause of death’ or ‘contributing factor’ included one or more of the following: ‘intoxication’, ‘toxicity’, ‘drug’, ‘medication’, ‘medicine’, ‘intake’, ‘narcot*’, ‘substance’, ‘intravenous’, ‘overdose’ or ‘poisoning’ by any drug (excluding carbon monoxide, cyanide or a non-drug substance). The UK data were drawn from the National Programme on Substance Abuse Deaths (NPSAD), an observational cohort that collects voluntarily submitted coronial data on deaths related to drugs from England, Wales, Northern Ireland and associated Islands (Isle of Man, Jersey and Guernsey).^15,16^ Deaths are included if the coronial verdict verifies the death as drug-related or if an alternative conclusion is reached but psychoactive drugs were implicated (e.g. suicides following cocaine use, road traffic collisions following cannabis use).^17^ A 10-year sample, contemporaneous with the US data, was selected for analysis from 1 January 2012 to 31 December 2021. Although there are differences in terminology between each country's coronial/medical examiner nomenclature, for the purpose of this study all deaths contained in either data-set are subsequently referred to as ‘drug-related deaths’.

**Table 1:** Sociodemographic characteristics of individuals who were housed and experiencing homelessness at the time of their drug-related death between 2012 and 2021 in the UK and USA

		UK		*P*^a^	USA		*P*^b^	*P*^c^
		Housed, *n* (%)	Homeless, *n* (%)		Housed, *n* (%)	Homeless, *n* (%)		
Total sample		19 279 (100.0)	680 (100.0)		20 453 (100.0)	3978 (100.0)		<0.001
Age, years	<18	209 (1.1)	2 (0.3)	<0.001	595 (2.9)	37 (0.9)	<0.001	<0.001
	19–29	2640 (13.7)	93 (13.7)		3858 (18.9)	390 (9.8)		
	30–39	5107 (26.5)	251 (36.9)		4391 (21.5)	763 (19.2)		
	40–49	6027 (31.5)	234 (34.4)		4101 (20.1)	891 (22.4)		
	50–59	3495 (18.1)	88 (12.9)		4511 (22.1)	1232 (31.0)		
	60–69	1175 (6.1)	12 (1.8)		2446 (12.0)	600 (15.1)		
	>70	581 (3.0)	0 (0.0)		521 (2.5)	60 (1.5)		
Sex	Female	5499 (28.5)	84 (12.4)	<0.001	5854 (28.6)	853 (21.4)	<0.001	<0.001
	Male	13 780 (71.5)	596 (87.6)		14 565 (71.2)	3117 (78.4)		
Ethnicity	White	10 746 (55.7)	439 (64.6)	0.001	10 316 (50.4)	1727 (43.4)	<0.001	<0.001
	Black	96 (0.5)	6 (0.9)		3486 (17.0)	1009 (25.4)		
	Asian	67 (0.3)	4 (0.6)		602 (2.9)	45 (1.1)		
	Other	159 (0.8)	4 (0.6)		5727 (28.0)	1132 (28.5)		
	Unknown	8211 (42.6)	227 (33.4)		322 (1.6)	65 (1.6)		
US county jurisdiction and date range	England	17 255 (89.5)	630 (92.7)	<0.001				
	Wales	716 (3.7)	31 (4.6)					
	Northern Ireland	1241 (6.4)	18 (2.7)					
	The Islands^d^	67 (0.4)	1 (0.2)					
	Maricopa County, Arizona (12/2019–03/2021)				1993 (9.7)	393 (9.9)	<0.001	
	Pinal County, Arizona (01/2017–12/2021)				406 (2.0)	34 (0.9)		
	Gila County, Arizona (01/2017–12/2021)				38 (0.2)	6 (0.2)		
	Los Angeles County, California (01/2012–12/2021)				13 721 (67.1)	3349 (84.2)		
	Denver County, Colorado (01/2019–12/2019)				174 (0.9)	33 (0.8)		
	Twin Falls County, Idaho (01/2016–12/2021)				118 (0.6)	1 (0.0)		
	Summit County, Ohio (01/2016–12/2021)				632 (3.1)	10 (0.3)		
	Harris County, Texas (06/2016–05/2021)				3371 (16.5)	152 (3.8)		

a. Comparing housed people with people experiencing homelessness in the UK.

b. Comparing housed people with people experiencing homelessness in the USA.

c. Comparing homeless people in the UK with homeless people in the USA.

d. The Isle of Man, Jersey and Guernsey.

NPSAD does not include records where the sole substance implicated was alcohol, and such deaths were therefore excluded *a priori* from the US data to ensure comparability. Drug-related deaths from each data-set were selected for analysis only if the coronial record contained information on housing status at the time of death. In both data-sets only binary variables were available, stating whether an individual was housed or homeless (or of ‘no fixed abode’ in the UK data) based on coronial/medical examiner records of whether the decedent had neither a temporary or permanent address and was considered unsheltered at the time of death. Both data-sets additionally contained information on individual decedents’ year of death, age at death, sex and which individual substances were implicated in death, i.e. those substances deemed causative of the death. These were derived from death certificate diagnoses – either when substances were specifically named (e.g. morphine toxicity) or, in cases where ambiguous causes are given (e.g. multidrug toxicity), taken from those listed as implicated in the toxicologist's interpretation of toxicology results, where available. The decedent's ethnicity was also available and categories were harmonised across data-sets to facilitate analysis (a description of ethnicity categories and harmonisation can be found in Supplementary Table 2).

### Statistical analysis

We initially described and compared the proportion of drug-related deaths between individuals who were housed and those who were homeless at the time of their death in both the USA and the UK, how they differ by sociodemographic characteristics (sex, age and ethnicity) and by individual substances identified as implicated in death. We additionally compared differences in drug-use profiles implicated in death, specified *a priori* as (a) only non-opioid substances implicated, (b) only opioids implicated and (c) any opioid plus any non-opioid substance implicated. Continuous variables were compared using an unpaired *t*-test, categorical variables using a chi-squared test and ordinal variables using the Wilcoxon rank sum test.

We additionally investigated for any association between an individual's housing status at the time of death and their drug-use profile. We developed a multilevel multinomial regression model with the exposure as the binary housing status at the time of death and the outcome as the three-level categorical variable of drug-use profile implicated in death.^18^ Individual decedents were more likely to share similarities if they were from the same coronial/medical examiner jurisdiction and thus the multilevel model accounted for clustering at the level of jurisdiction and was adjusted for all available socioeconomic characteristics (age, sex and ethnicity) and year of death.

To test whether drug-use profiles implicated in death were the same for the UK and the USA we developed a stacked model and used a chi-squared test to assess whether the intercepts in the US and UK multilevel multinomial regression models were the same.^19,20^ If they were we planned to proceed with a pooled model for both countries and if not we planned to test for differences within each individual country's sample. All analyses were conducted in STATA IC version 15 for Windows, with the significance level set at 0.05; no adjustments were made for multiple testing. Where analyses were not described in the pre-registered analysis plan and are reported only in the Supplementary material these should be considered exploratory.

### Ethics statement

The authors assert that all procedures contributing to this work comply with the ethical standards of the relevant national and institutional committees on human experimentation and with the Helsinki Declaration of 1975, as revised in 2008. The King's College London (KCL) Biomedical and Health Sciences, Dentistry, Medicine and Natural and Mathematical Sciences Research Ethics Subcommittee (BDM RESC) re-confirmed in August 2022 that NPSAD does not require Research Ethics Committee (REC) review and the Institutional Review Board (IRB) at UCLA confirmed in November 2021 that the US data-set is exempt from IRB approval, as all individuals are deceased.

## Results

The total number of drug-related deaths during the studied time frame was *n* = 20 061 in the UK data-set and *n* = 24 554 in the US data-set. Housing status at time of death was available for *n* = 19 959 (99.5%) and *n* = 24 431 (99.5%) respectively. These made up the final included samples, of which *n* = 680 (3.4%) people were experiencing homelessness at the time of death in the UK, compared with *n* = 3978 (16.3%) in the USA (*P* < 0.001).

### Inter- and intra-country socioeconomic characteristics

A breakdown of socioeconomic and jurisdiction characteristics by housing status and country can be found in Table 1. In both countries, homeless decedents were more likely to include higher proportions of people identifying as male (87.6% *v.* 71.5%, *P* < 0.001 (UK); 78.4% *v.* 71.2%, *P* < 0.001 (USA)).

In the UK, homeless decedents were significantly younger compared with those in the USA (*P* < 0.001), the majority of UK homeless decedents being aged between 30–49 years at death, compared with 40–59 years for the majority of US homeless decedents. In the UK, homeless decedents had significantly higher proportions identified as of White ethnicity compared with the USA, homeless decedents in the USA having significantly higher proportions identified as of Black ethnicity (*P* < 0.001). However, in the UK over two-fifths of all decedents (42.3%) had no recorded ethnicity.

### Inter- and intra-country specific substances implicated in death

A breakdown of individual drugs and drug-use profiles implicated in death by housing status and country can be found in Table 2. In both countries, homeless and housed decedents had a similar overall mean number of substances implicated in death (2.8 *v.* 2.8, *P* = 0.5 (UK); 1.5 *v.* 1.6, *P* = 0.13 (USA)), although homeless decedents in the UK had a significantly higher overall mean number of substances implicated in death compared with those in the USA (2.8 *v.* 1.5; *P* < 0.001). In both countries, the only substances implicated in death in a significantly lower proportion of homeless decedents compared with housed individuals were oxycodone (1.2% *v.* 2.5%, *P* = 0.02 (UK); 0.6% *v.* 3.9%, *P* < 0.001 (USA)) and gabapentin (1.9% *v.* 3.4%, *P* = 0.04 (UK); 0.2% *v.* 1.0%, *P* < 0.001 (USA)). However, 1.9% of homeless decedents in the UK had gabapentin implicated in death, compared with only 0.2% in the USA (*P* < 0.001).

**Table 2:** Drugs implicated in deaths of individuals who were housed and experiencing homelessness at the time of their drug-related death between 2012 and 2021 in the UK and USA

	Drug	UK		*P*^a^	USA		*P*^b^	*P*^c^
		Housed, *n* (%)	Homeless, *n* (%)		Housed, *n* (%)	Homeless, *n* (%)		
All		19 279 (100.0)	680 (100.0)	–	20 453 (100.0)	3978 (100.0)	–	
Opioid implicated	Any opioid(s) implicated	12 682 (65.8)	547 (80.4)	<0.001	10 595 (51.8)	1727 (43.4)	<0.001	<0.001
	Only opioid(s) implicated	3424 (17.8)	138 (20.3)	0.09	4891 (23.9)	427 (10.7)	<0.001	<0.001
	Heroin	7911 (41.0)	431 (63.4)	<0.001	2858 (14.0)	581 (14.6)	0.3	<0.001
	Methadone	3650 (18.9)	164 (24.1)	<0.001	585 (2.9)	84 (2.1)	0.01	<0.001
	Buprenorphine	328 (1.7)	11 (1.6)	0.87	40 (0.2)	4 (0.1)	0.20	<0.001
	Codeine	1787 (9.3)	44 (6.5)	0.01	219 (1.1)	12 (0.3)	<0.001	<0.001
	Oxycodone	488 (2.5)	8 (1.2)	0.02	789 (3.9)	22 (0.6)	<0.001	0.06
	Any type of fentanyl^d^	443 (2.3)	9 (1.3)	0.93	5800 (28.4)	1051 (26.4)	0.01	<0.001
Non-opioid substance implicated (excluding alcohol)	Any non-opioid substance/s implicated	15 855 (82.2)	542 (79.7)	0.09	12 808 (62.6)	3262 (82.0)	<0.001	0.18
	Only non-opioid substance(s) implicated	6597 (34.2)	133 (19.6)	<0.001	7104 (34.7)	1962 (49.3)	<0.001	<0.001
	Any benzodiazepine(s) implicated	3900 (20.2)	148 (21.8)	0.33	2298 (11.2)	99 (2.5)	<0.001	<0.001
	Only benzodiazepine(s) implicated	32 (0.2)	0 (0.0)	0.30	250 (1.2)	10 (0.3)	<0.001	0.19
	Alprazolam	382 (2.0)	13 (1.9)	0.90	1464 (7.2)	40 (1.0)	<0.001	0.04
	Methamphetamine	74 (0.4)	3 (0.4)	0.81	7442 (36.4)	2654 (66.7)	<0.001	<0.001
	Cocaine^e^	3681 (19.1)	134 (19.7)	0.69	4263 (20.8)	682 (17.1)	<0.001	0.10
	Gabapentin	652 (3.4)	13 (1.9)	0.04	207 (1.0)	7 (0.2)	<0.001	<0.001
	Any synthetic cannabinoid receptor agonist(s) (SCRA) implicated	224 (1.2)	37 (5.4)	<0.001	32 (0.2)	4 (0.1)	<0.001	<0.001
	Only synthetic cannabinoid receptor agonist(s) (SCRA) implicated	89 (0.5)	18 (2.7)	<0.001	27 (0.1)	2 (0.1)	0.17	<0.001
Any opioid plus any non-opioid implicated	Any opioid(s) + Any non-opioid substance(s) implicated	9326 (48.4)	410 (60.3)	<0.001	5704 (27.9)	1300 (32.7)	<0.001	<0.001
Total number of drugs implicated, mean (s.d.)		2.8 (1.8)	2.8 (1.9)	0.50	1.6 (0.8)	1.5 (0.7)	0.13	<0.001

a. Comparing housed people with people experiencing homelessness in the UK.

b. Comparing housed people with people experiencing homelessness in the USA.

c. Comparing with homeless people in the UK with people experiencing homelessness in the USA.

d. Includes only fentanyl or any type of fentanyl analogue.

e. Includes both crack and powder cocaine.

Interestingly, almost all significant differences comparing which individual substances were implicated in death in homeless decedents in the UK were the inverse of those in the USA, with a significantly a higher proportion having any opioid implicated in death (homeless *v*. housed: 80.4% *v.* 65.8%, *P* < 0.001 (UK); 43.4% *v.* 51.8%, *P* < 0.001 (USA)), methadone implicated (24.1% *v.* 18.9%, *P* < 0.001 (UK); 2.1% *v.* 2.9%, *P* = 0.01 (USA)) and any synthetic cannabinoid receptor agonist (SCRA) implicated (5.4% *v.* 1.2%, *P* < 0.001 (UK); 0.1% *v.* 0.2%, *P* < 0.001 (USA)), and a significantly lower proportion had only non-opioid substances implicated (19.6% *v.* 34.2%, *P* < 0.001 (UK); 49.3% *v.* 34.7%, *P* < 0.001 (USA)).

Fentanyl and fentanyl analogues were implicated in the deaths of over a quarter of all homeless decedents in the USA (26.4%) and methamphetamine implicated in over two-thirds (66.7%). A significantly higher proportion of homeless decedents in the USA had methamphetamine implicated in death compared with housed individuals (66.7% *v.* 36.4%, *P* < 0.001), with the proportion of homeless decedents in the US having ninety-five-fold the rate of methamphetamine implicated in death compared with UK homeless decedents (66.7% *v.* 0.4%, *P* < 0.001). Trends by year of death for each specific substance implicated in death are available in Supplementary Table 3.

### Inter- and intra-country specific drug-use profiles implicated in death

In terms of the pre-specified drug-use profiles, compared with housed individuals, homeless decedents in the UK were significantly (*P* < 0.001) less likely to have (a) only non-opioid substances implicated in death but significantly more likely to have (b) only opioids implicated and (c) any opioid plus any non-opioid substance implicated. Conversely in the USA, compared with housed individuals, homeless decedents were significantly (*P* < 0.001) more likely to have (a) only non-opioid substances implicated and significantly less likely to have (b) only opioids implicated.

The stacked multilevel multinomial regression models demonstrated strong evidence (*P* < 0.001) that the intercepts of the UK and US models were significantly different and we therefore developed two individual models to assess differences within each country's sample. The results of the multilevel multinomial regression can be found in Table 3. Following adjustment there was strong evidence that among people dying due to drug-related causes in the UK, when compared with housed individuals, homeless decedents were significantly more likely to have (a) only opioids implicated in death (relative risk ratio RRR = 1.87, 95% CI 1.76–1.98, *P* < 0.001) and (b) both opioid and non-opioid substances implicated in death (RRR = 2.04, 95% CI 1.84–2.27, *P* < 0.001) relative to having only non-opioid substances implicated. Conversely, following adjustment there was strong evidence that among people dying due to drug-related causes in the USA, homeless decedents were significantly less likely to have only opioids implicated in death (RRR = 0.37, 95% CI 0.29–0.48, *P* < 0.001) relative to having only non-opioid substances implicated. Data from the USA were insensitive to any difference when comparing whether both opioid and non-opioid substances were implicated (RRR = 0.90, 95% CI 0.71–1.14, *P* = 0.36) relative to having only non-opioid substances implicated.

**Table 3:** Relative risk of drug-related death for specific drug use profiles implicated in death between 2012 and 2021 in the UK and USA^a^

			UK		USA	
			RRR (95% CI)	*P*	RRR (95% CI)	*P*
Only opioid implicated	Housing status at time of death	Housed	Reference			
		Homeless	1.87 (1.76–1.98)	<0.001	0.37 (0.29–0.48)	<0.001
	Age, years	<18	Reference			
		19–29	1.82 (1.45–2.27)	<0.001	2.75 (1.64–4.60)	<0.001
		30–39	2.72 (2.18–3.40)	<0.001	1.19 (0.67–2.14)	0.55
		40–49	2.89 (2.32–3.59)	<0.001	0.59 (0.28–1.27)	0.18
		50–59	2.60 (2.05–3.30)	<0.001	0.48 (0.18–1.29)	0.14
		60–69	2.50 (1.97–3.18)	<0.001	0.58 (0.20–1.71)	0.33
		>70	2.78 (2.19–3.54)	<0.001	0.81 (0.28–2.38)	0.71
	Gender	Female	Reference			
		Male	1.20 (1.11–1.29)	<0.001	0.98 (0.88–1.10)	0.79
	Ethnicity	White	Reference			
		Black	0.40 (0.34–0.45)	<0.001	0.31 (0.23–0.41)	<0.001
		Asian	1.02 (0.89–1.17)	0.75	0.31 (0.27–0.36)	<0.001
		Other	1.02 (1.02–1.03)	<0.001	0.61 (0.51–0.73)	<0.001
		Unknown	0.88 (0.81–0.96)	0.003	0.43 (0.35–0.53)	<0.001
	Year of death	2012–2013	Reference			
		2014–2015	1.43 (1.32–1.55)	<0.001	0.74 (0.73–0.74)	<0.001
		2016–2017	1.41 (1.30–1.54)	<0.001	0.67 (0.61–0.74)	<0.001
		2018–2019	1.25 (1.10–1.43)	0.001	0.70 (0.60–0.81)	<0.001
		2020–2021	1.09 (0.86–1.38)	0.47	0.93 (0.90–0.96)	<0.001
Opioid + non-opioid implicated	Housing status at time of death	Housed	Reference			
		Homeless	2.04 (1.84–2.27)	<0.001	0.90 (0.71–1.14)	0.36
	Age, years	<18	Reference			
		19–29	2.45 (2.30–2.62)	<0.001	5.48 (4.82–6.23)	<0.001
		30–39	4.25 (3.86–4.68)	<0.001	2.95 (2.30–3.78)	<0.001
		40–49	4.60 (3.86–5.48)	<0.001	1.61 (1.32–1.97)	<0.001
		50–59	3.81 (3.51–4.13)	<0.001	1.12 (1.03–1.20)	0.005
		60–69	2.61 (2.30–2.96)	<0.001	1.03 (0.91–1.23)	0.66
		>70	1.70 (1.50–1.92)	<0.001	0.71 (0.65–0.78)	<0.001
	Gender	Female	Reference			
		Male	1.03 (0.98–1.08)	0.38	0.90 (0.70–1.13)	0.35
	Ethnicity	White	Reference			
		Black	0.35 (0.34–0.35)	<0.001	0.40 (0.32–0.49)	<0.001
		Asian	0.66 (0.58–0.75)	<0.001	0.37 (0.30–0.47)	<0.001
		Other	0.54 (0.52–0.55)	<0.001	0.54 (0.48–0.61)	<0.001
		Unknown	0.90 (0.83–0.97)	0.007	0.54 (0.45–0.66)	<0.001
	Year of death	2012–2013	Reference			
		2014–2015	1.27 (1.17–1.37)	<0.001	1.32 (1.32–1.33)	<0.001
		2016–2017	1.34 (1.29–1.40)	<0.001	1.97 (1.42–2.74)	<0.001
		2018–2019	1.37 (1.33–1.42)	<0.001	2.66 (1.99–3.57)	<0.001
		2020–2021	1.36 (1.25–1.47)	<0.001	4.91 (4.71–5.13)	<0.001

RRR, relative risk ratio.

a. Model reference category is compared to individuals with only non-opioid substances implicated in death.

## Discussion

Despite a similar overall number of substances being implicated in death among those dying due to drug-related causes in the UK and the USA, there were significant differences in drug-use profile both intra-country when comparing their housed and homeless populations, and inter-country when comparing each country's homeless population.

In the UK, opioids were implicated in two-thirds (66.3%) of all studied drug-related deaths. When compared with housed individuals, people experiencing homelessness were at a 1.5- to 2-fold increased risk of having only opioids implicated in death, relative to having only non-opioids implicated. Conversely in the USA, opioids were implicated in half (50.4%) of all studied drug-related deaths. When compared with housed individuals, people experiencing homelessness were at a 2- to 3-fold decreased risk of having only opioids implicated in death, relative to having only non-opioids implicated. In over two-thirds of the homeless decedents in the USA methamphetamine was implicated in death, compared with less than one in a hundred in the UK.

### Targeted drug treatment interventions

Despite both countries currently experiencing unprecedented numbers of drug-related deaths,^1,2^ with disproportionate drug-related harms experienced among their homeless populations,^3^ the results suggest that the suite of drug treatment interventions targeted at homeless populations in each country is likely to require differential prioritisation. In the UK it would appear prudent that exquisite focus is placed on identification of any opioid use among people experiencing homelessness and support should be provided to access evidence-based interventions to reduce the risk of opioid overdose, including, but not limited to, ensuring the provision and availability of take-home naloxone and reducing barriers to accessing opioid agonist treatment.^21,22^ Conversely prioritisation of evidence-based interventions to reduce or prevent methamphetamine use – such as contingency management and pharmacotherapy with emerging evidence of effectiveness – among the population experiencing homelessness in the USA would appear more targeted, and the results may suggest that take home-naloxone programmes, although critical, may be insufficient to combat the magnitude of drug-related deaths within the homeless population in the studied areas of the USA.^23,24^ These observations may also assist design of homelessness services, with self-reported drug use or point-of-care testing that show drug profiles associated with increased risk triggering prioritisation, assertive outreach and adequate support and follow-up.

### Differing prevalence of homelessness

Although the prevalence of homelessness is not static in any geographical region, and its magnitude at any point in time is subject to several factors, including housing availability and current economic climate,^25^ the prevalence of homelessness in the studied data-sets differs significantly between each country (3.4% in the UK *v.* 16.3% in the USA, *P* < 0.001). Although this is, in part, reflective of overall homeless population estimates within both countries,^26,27^ it is also the case that the harder it is to become homeless in a region is correlated with increased complexity in the health and social care needs of individuals who do become homeless.^28^ One might therefore expect individuals to have more severe drug-related problems in jurisdictions in which homelessness is rarer, whereas the societal burden of drug problems may be greater in jurisdictions where homelessness is more common, given the bidirectional relationship between drug use and homelessness.^3,25^ Internationally comparative studies may therefore have specific utility in demonstrating inter-country differences and also provide weight to argue that both drug and non-drug social welfare policies are likely to affect drug-related outcomes, including death, differentially in differing regions’ homeless populations. The UK has a more generous social safety net, including housing and unemployment support, compared with the USA. Not only does this contribute to a lower overall homelessness prevalence, but also it means that, on average, the people who do ultimately experience homelessness in the UK are likely to face more health and social challenges than their US counterparts. This is relevant as any interventions targeted at mitigating poor outcomes within these populations need to be aware of differing pathways into homelessness when drawing conclusions, particularly if combining research in reviews and/or meta-analyses. For example, Housing First programmes might work well in places where, for many people, the only problem is housing but not generalise to places where people experiencing homelessness have, on average, more severe health and social care needs.

### Strengths, limitations and future research

There are significant strengths to the study, including pre-registration of the study protocol and standardised reporting, with the detail of individual substances implicated in death providing a more granular understanding of drug profile implicated in death. This is also the first study, to our knowledge, to look comparatively at large contemporaneous international samples of drug-related deaths among homeless populations.

There are a number of limitations. The binary coding of homelessness status in both data-sets fails to account for the complexity and spectrum of homelessness and those at risk of homelessness, with dichotomous categorisations justly discouraged in research.^29^ Both data-sets probably underestimate the number of people in temporary or insecure housing, and estimates of increased risk within homeless populations may therefore be conservative. However, as few publicly available data-sets on drug-related harm routinely capture any housing status metric there remains utility in using this binary categorisation to develop a preliminary understanding of the epidemiological profile within this cohort. The data from the USA come from only eight jurisdictions and thus should not be considered representative of all US drug-related deaths; indeed, there is a relatively lower percentage of drug-related deaths with opioids implicated in the US sample (50.4%) compared with whole-country estimates of the proportion of drug overdose deaths in which an opioid was involved (75%),^2^ probably reflecting the western geographical skew in the available studied jurisdictions and the region of the country with the most deeply entrenched methamphetamine problem.^30^ However, several large-scale publicly available data-sets used to assess drug-related harm (e.g. CDC WONDER in the USA and the Office for National Statistics’ annual reports on drug poisoning in the UK)^1,8^ do not routinely collect or report housing status. We also note that Scotland ceased reporting to NPSAD in 2011 and therefore data were not available for that country. Despite these limitations, with no large-scale data-sets available, the current sample retains utility to explore outcomes within the understudied homeless populations of the USA and UK, and given the differential profiles observed, our results serve to highlight a need for the spectrum of housing status to be routinely recorded and explored in larger samples.

Additionally, the voluntary nature of data submission and differential access to toxicology testing across each country and jurisdiction is likely to lead to variation in results on the overall number of substances and which are deemed implicated in death. Although larger, representative samples in the USA would further help establish local or regional patterns that might aid local practitioners in targeting specific population needs or monitoring local drug trends, there remains utility in examining differences in a broad cohort of drug-related deaths. Unfortunately, without routine collection of housing status in US death investigations or national statistics, it is not currently possible for researchers to analyse the impact of housing across the whole (or even most) of the national population. However, given the above, and the significant difference in regression model intercepts, which prevents pooling of the two countries’ data,^19^ broad international comparisons should be interpreted with caution. Intra-country results are likely to have more utility in relation to each country's drug-related policy and practice, but the comparison highlights the potential roles of macro-level factors that may be difficult to recognise without looking outside a country's own context. The 10-year sampling frame, the national nature of the UK data, and sampling multiple urban and rural US jurisdictions partially mitigates these concerns. However, we acknowledge that neither data-set contains data subsequent to December 2021, during which period the COVID-19 pandemic continued to dramatically affect homelessness and personal circumstances, which may increase the risk of homelessness.^21^ This study does not comment on changes that may have occurred due to COVID-19 but, given the disproportionate impact the pandemic had on marginalised communities, inequalities in drug-related deaths may have followed similar trajectories and continued monitoring is an avenue for further study. Across both data-sets we are also unable to accurately ascertain and adjust for which decedents were accessing evidenced-based treatment at the time of their death, so data linkage to treatment records may be another fruitful avenue for further study. Last, although our pre-specified significance level of 0.05 did not adjust for multiple testing, *post hoc* Bonferroni corrections did not alter the significance of any results from pre-registered hypotheses.

### Implications

Given the significant differential drug use and risk profiles demonstrated in this marginalised population, routine data capture of housing status would enable trend monitoring, including the response to any specific interventions, and potentially aid targeting of treatments at this vulnerable population to improve health equity. Given the substantial burden of drug-related death within populations experiencing homelessness, this study adds evidence to advocate for housing status to be routinely recorded, a move that could also facilitate exploration of any nuances in drug-related harms across the spectrum of housing insecurity. Homeless populations experience drug harms differently than housed individuals with demonstrated differential drug use profiles and associated different risks of drug-related death. When considering each country’s homeless population dying due to drug-related causes, opioid involvement appears key in the UK with methamphetamine a significant burden in the studied US jurisdictions. Practitioners should be diligent about assessment and monitoring of drug use within these populations to ensure appropriate preventive, treatment and harm-reduction interventions that are specifically targeted at people experiencing homelessness.

## Supporting information

Roberts et al. supplementary material Roberts et al. supplementary material

## Data Availability

The data that support the findings of this study are available from the corresponding author on reasonable request, subject to institutional and ethical approval. The analytic code is also available from the corresponding author on reasonable request.
