# Supplementary material for: Drug-related deaths among housed and homeless individuals in the UK and the USA: comparative retrospective cohort study
Source: Br J Psychiatry. 2023 Dec;223(6):562–8. doi: 10.1192/bjp.2023.111 (PMC10727910; doi:10.1192/bjp.2023.111)
Supplement: Roberts et al. supplementary material [file S0007125023001113sup001.docx]

**Online Supplementary Material**

Contents

**Tables**

S1. STrengthening the Reporting of OBservational studies in Epidemiology (STROBE) Checklist for cohort studies (1)

S2. Harmonised ethnicity categorisation

S3. Drugs implicated in death comparing decedents who were housed and those experiencing homelessness at the time of their death due to drug-related causes between 2012 - 2021 in the United Kingdom and the United States: A breakdown by year of death

**References**

**Tables**

Table S1: STrengthening the Reporting of OBservational studies in Epidemiology (STROBE) Checklist for cohort studies (1)

|  | Item No | Recommendation |
| --- | --- | --- |
| **Title and abstract** | 1 | (*a*) Indicate the study’s design with a commonly used term in the title or the abstract; Title: Page 1 |
|  |  | (*b*) Provide in the abstract an informative and balanced summary of what was done and what was found; Abstract: Page 3 |
| Introduction | | |
| Background/rationale | 2 | Explain the scientific background and rationale for the investigation being reported; Introduction: Page 4/5 |
| Objectives | 3 | State specific objectives, including any prespecified hypotheses; Introduction: Page 5 |
| Methods | | |
| Study design | 4 | Present key elements of study design early in the paper; Methods: Page 6 |
| Setting | 5 | Describe the setting, locations, and relevant dates, including periods of recruitment, exposure, follow-up, and data collection; Methods: Pages 6/7 |
| Participants | 6 | (*a*) Give the eligibility criteria, and the sources and methods of selection of participants. Describe methods of follow-up; Methods: Pages 6/7 |
|  |  | (*b*) For matched studies, give matching criteria and number of exposed and unexposed N/A |
| Variables | 7 | Clearly define all outcomes, exposures, predictors, potential confounders, and effect modifiers. Give diagnostic criteria, if applicable Methods: Page 7/8 |
| Data sources/ measurement | 8* | For each variable of interest, give sources of data and details of methods of assessment (measurement). Describe comparability of assessment methods if there is more than one group Methods: Page 7/8 |
| Bias | 9 | Describe any efforts to address potential sources of bias Methods: Page 7/8 |
| Study size | 10 | Explain how the study size was arrived at Methods Page 6/7 ; Results Page 9 |
| Quantitative variables | 11 | Explain how quantitative variables were handled in the analyses. If applicable, describe which groupings were chosen and why Methods Page 7/8 |
| Statistical methods | 12 | (*a*) Describe all statistical methods, including those used to control for confounding Methods Page 7/8 |
|  |  | (*b*) Describe any methods used to examine subgroups and interactions Methods Page 7/8 |
|  |  | (*c*) Explain how missing data were addressed Methods Page 7 |
|  |  | (*d*) If applicable, explain how loss to follow-up was addressed N/A |
|  |  | (*e*) Describe any sensitivity analyses Methods Page N/A |
| Results | | |
| Participants | 13* | (a) Report numbers of individuals at each stage of study—eg numbers potentially eligible, examined for eligibility, confirmed eligible, included in the study, completing follow-up, and analysed Results Page 9 |
|  |  | (b) Give reasons for non-participation at each stage Results Page N/A |
|  |  | (c) Consider use of a flow diagram N/A |
| Descriptive data | 14* | (a) Give characteristics of study participants (eg demographic, clinical, social) and information on exposures and potential confounders Results Pages 9 (Table 1) |
|  |  | (b) Indicate number of participants with missing data for each variable of interest Table 1 |
|  |  | (c) Summarise follow-up time (eg, average and total amount) Methods Page 6/7 |
| Outcome data | 15* | Report numbers of outcome events or summary measures over time Table 9/10/11 |
| Main results | 16 | (*a*) Give unadjusted estimates and, if applicable, confounder-adjusted estimates and their precision (eg, 95% confidence interval). Make clear which confounders were adjusted for and why they were included Table 1/2/3 |
|  |  | (*b*) Report category boundaries when continuous variables were categorized Table 1/2 |
|  |  | (*c*) If relevant, consider translating estimates of relative risk into absolute risk for a meaningful time period N/A |
| Other analyses | 17 | Report other analyses done—eg analyses of subgroups and interactions, and sensitivity analyses Table 3 Results Pages 10/11 |
| Discussion | | |
| Key results | 18 | Summarise key results with reference to study objectives Discussion Page 12 |
| Limitations | 19 | Discuss limitations of the study, taking into account sources of potential bias or imprecision. Discuss both direction and magnitude of any potential bias Discussion Page 13/14 |
| Interpretation | 20 | Give a cautious overall interpretation of results considering objectives, limitations, multiplicity of analyses, results from similar studies, and other relevant evidence Conclusions Page 16 |
| Generalisability | 21 | Discuss the generalisability (external validity) of the study results Discussion Page 15/16 |
| Other information | | |
| Funding | 22 | Give the source of funding and the role of the funders for the present study and, if applicable, for the original study on which the present article is based Methods Page 2 |

S2: Harmonised ethnicity categorisation

| **United Kingdom** | **United States of America** | **Harmonised** |
| --- | --- | --- |
| White | White | White |
| Black Caribbean | Black | Black |
| Black African |  |  |
| Black Other |  |  |
| Indian | Asian | Asian |
| Pakistani |  |  |
| Bangladeshi |  |  |
| Chinese |  |  |
| Other | American Indian or Alaskan Native | Other |
|  | Native Hawaiian and other Pacific Islander |  |
|  | Hispanic/Latin American |  |
|  | Other |  |
| Unknown | Unknown | Unknown |

S3: Drugs implicated in death comparing decedents who were housed and those experiencing homelessness at the time of their death due to drug-related causes between 2012 - 2021 in the United Kingdom and the United States: A breakdown by year of death

|  | | United Kingdom | | United States | |
| --- | --- | --- | --- | --- | --- |
|  |  | Housed  n (%) | Experiencing Homelessness n (%) | Housed  n (%) | Experiencing Homelessness n (%) |
| Any opioid/s implicated | All | 12682 (100.0) | 547 (100.0) | 10595 (100.0) | 1727 (100.0) |
|  | 2012/13 | 1884 (14.9) | 83 (15.2) | 619 (5.8) | 64 (3.7) |
|  | 2014/15 | 2347 (18.5) | 99 (18.1) | 716 (6.8) | 95 (5.5) |
|  | 2016/17 | 2461 (19.4) | 119 (21.8) | 1377 (13.0) | 176 (10.2) |
|  | 2018/19 | 2934 (23.1) | 143 (26.1) | 2313 (13.8) | 278 (16.1) |
|  | 2020/21 | 3056 (24.1) | 103 (18.8) | 5323 (50.2) | 1051 (60.9) |
| Only opioid/s implicated | All | 3424 (100.0) | 138 (100.0) | 4891 (100.0) | 427 (100.0) |
|  | 2012/13 | 526 (15.4) | 24 (17.4) | 458 (9.4) | 48 (11.2) |
|  | 2014/15 | 706 (20.6) | 30 (21.7) | 453 (9.3) | 45 (10.5) |
|  | 2016/17 | 721 (21.1) | 26 (18.8) | 640 (13.1) | 67 (15.7) |
|  | 2018/19 | 756 (22.1) | 36 (26.1) | 990 (20.2) | 69 (16.2) |
|  | 2020/21 | 715 (20.9) | 22 (15.9) | 2245 (45.9) | 189 (44.3) |
| Heroin | All | 7911 (100.0) | 431 (100.0) | 2858 (100.0) | 581 (100.0) |
|  | 2012/13 | 983 (12.4) | 58 (13.5) | 302 (10.6) | 47 (8.1) |
|  | 2014/15 | 1517 (19.9) | 85 (19.7) | 311 (10.9) | 60 (10.3) |
|  | 2016/17 | 1599 (20.2) | 103 (23.9) | 534 (18.7) | 115 (19.8) |
|  | 2018/19 | 1940 (24.5) | 113 (26.2) | 838 (29.3) | 153 (26.3) |
|  | 2020/21 | 1818 (23.0) | 72 (16.7) | 843 (29.5) | 204 (35.1) |
| Methadone | All | 3650 (100.0) | 164 (100.0) | 585 (100.0) | 84 (100.0) |
|  | 2012/13 | 590 (16.2) | 25 (15.2) | 88 (15.0) | 5 (6.0) |
|  | 2014/15 | 604 (16.6) | 18 (11.0) | 85 (14.5) | 14 (16.7) |
|  | 2016/17 | 608 (16.7) | 29 (17.7) | 125 (21.4) | 22 (26.2) |
|  | 2018/19 | 744 (20.4) | 54 (32.9) | 118 (20.2) | 15 (17.9) |
|  | 2020/21 | 1104 (30.3) | 38 (23.2) | 163 (27.9) | 27 (32.1) |
| Buprenorphine | All | 328 (100.0) | 11 (100.00) | 40 (100.0) | 4 (100.0) |
|  | 2012/13 | 24 (7.3) | 4 (36.4) | 0 (0.0) | 0 (0.0) |
|  | 2014/15 | 44 (13.4) | 1 (9.1) | 1 (2.5) | 1 (25.0) |
|  | 2016/17 | 54 (16.5) | 0 (0.0) | 8 (20.0) | 0 (0.0) |
|  | 2018/19 | 83 (25.3) | 3 (27.3) | 6 (15.0) | 3 (75.0) |
|  | 2020/21 | 123 (37.5) | 3 (27.3) | 25 (62.5) | 0 (0.0) |
| Codeine | All | 1787 (100.0) | 44 (100.0) | 219 (100.0) | 12 (100.0) |
|  | 2012/13 | 354 (19.8) | 11 (25.0) | 5 (2.3) | 0 (0.0) |
|  | 2014/15 | 382 (21.4) | 8 (18.2) | 15 (6.9) | 1 (8.3) |
|  | 2016/17 | 340 (19.0) | 8 (18.2) | 67 (30.6) | 3 (25.0) |
|  | 2018/19 | 377 (21.1) | 10 (22.7) | 69 (31.5) | 3 (25.0) |
|  | 2020/21 | 33 (18.7) | 7 (15.9) | 61 (27.9) | 5 (41.7) |
| Oxycodone | All | 488 (100.0) | 8 (100.0) | 789 (100.0) | 22 (100.0) |
|  | 2012/13 | 70 (14.3) | 2 (25.0) | 48 (6.1) | 2 (9.1) |
|  | 2014/15 | 91 (18.7) | 0 (0.0) | 95 (12.0) | 2 (9.1) |
|  | 2016/17 | 107 (21.9) | 1 (12.5) | 146 (18.5) | 5 (22.7) |
|  | 2018/19 | 111 (22.8) | 2 (25.0) | 199 (25.2) | 5 (22.7) |
|  | 2020/21 | 109 (22.3) | 3 (37.5) | 295 (37.4) | 7 (31.8) |
| Any type of fentanyl | All | 443 (100.0) | 9 (100.0) | 5800 (100.0) | 1051 (100.0) |
|  | 2012/13 | 43 (9.7) | 2 (22.2) | 18 (0.3) | 1 (0.1) |
|  | 2014/15 | 64 (14.5) | 1 (11.1) | 30 (0.5) | 1 (0.1) |
|  | 2016/17 | 153 (34.5) | 5 (55.5) | 275 (4.7) | 14 (1.3) |
|  | 2018/19 | 110 (24.8) | 1 (11.1) | 1060 (18.3) | 99 (9.4) |
|  | 2020/21 | 73 (16.5) | 0 (0.0) | 4205 (72.5) | 878 (93.5) |
| Any non-opioid substance/s implicated | All | 15855 (100.0) | 542 (100.0) | 12808 (100.0) | 3262 (100.0) |
|  | 2012/13 | 2644 (16.6) | 68 (12.5) | 820 (6.4) | 123 (3.8) |
|  | 2014/15 | 2830 (17.8) | 90 (16.5) | 1094 (8.5) | 213 (6.5) |
|  | 2016/17 | 2941 (18.5) | 123 (22.7) | 2100 (16.4) | 420 (12.9) |
|  | 2018/19 | 3613 (22.7) | 149 (27.4) | 3060 (23.9) | 725 (22.2) |
|  | 2020/21 | 3895 (24.5) | 112 (20.6) | 5456 (42.6) | 1647 (51.3) |
| Only non-opioid substance/s implicated | All | 6597 (100.0) | 133 (100.0) | 7104 (100.0) | 1962 (100.0) |
|  | 2012/13 | 1273 (19.3) | 9 (6.8) | 659 (9.3) | 107 (5.5) |
|  | 2014/15 | 1176 (17.8) | 21 (15.8) | 831 (11.7) | 163 (8.3) |
|  | 2016/17 | 1180 (17.9) | 30 (22.6) | 1363 (19.2) | 311 (15.9) |
|  | 2018/19 | 1423 (21.6) | 42 (31.6) | 1737 (24.5) | 516 (26.3) |
|  | 2020/21 | 1545 (23.4) | 31 (23.3) | 2378 (33.5) | 812 (41.4) |
| Any benzodiazepine/s implicated | All | 3900 (100.0) | 148 (100.0) | 2298 (100.0) | 99 (100.0) |
|  | 2012/13 | 532 (13.6) | 15 (10.1) | 46 (2.0) | 2 (2.0) |
|  | 2014/15 | 676 (17.3) | 20 (13.5) | 102 (4.4) | 4 (4.0) |
|  | 2016/17 | 726 (18.6) | 38 (25.7) | 434 (18.9) | 14 (14.1) |
|  | 2018/19 | 953 (24.4) | 40 (27.0) | 612 (26.6) | 22 (22.2) |
|  | 2020/21 | 1013 (26.0) | 35 (23.6) | 1060 (46.1) | 51 (51.5) |
| Only benzodiazepine/s implicated | All | 32 | 0 (0.0) | 250 (100.0) | 10 (100.0) |
|  | 2012/13 | 5 (15.6) | 0 (0.0) | 18 (7.2) | 0 (0.0) |
|  | 2014/15 | 5 (15.6) | 0 (0.0) | 21 (8.4) | 0 (0.0) |
|  | 2016/17 | 2 (6.2) | 0 (0.0) | 61 (24.4) | 2 (20.0) |
|  | 2018/19 | 6 (18.8) | 0 (0.0) | 69 (27.6) | 2 (20.0) |
|  | 2020/21 | 14 (43.8) | 0 (0.0) | 74 (29.6) | 6 (60.0) |
| Alprazolam | All | 382 (100.0) | 13 (100.0) | 1464 (100.0) | 40 (100.0) |
|  | 2012/13 | 9 (2.4) | 0 (0.0) | 22 (1.5) | 0 (0.0) |
|  | 2014/15 | 11 (2.9) | 1 (7.7) | 52 (3.6) | 0 (0.0) |
|  | 2016/17 | 98 (25.7) | 3 (23.1) | 274 (18.7) | 9 (22.5) |
|  | 2018/19 | 206 (53.9) | 8 (61.5) | 347 (24.4) | 8 (20.0) |
|  | 2020/21 | 58 (15.2) | 1 (7.7) | 729 (49.8) | 22 (55.0) |
| Methamphetamine | All | 74 (100.0) | 3 (100.0) | 7442 (100.0) | 2654 (100.0) |
|  | 2012/13 | 9 (12.2) | 0 (0.0) | 480 (6.5) | 81 (3.1) |
|  | 2014/15 | 12 (16.2) | 1 (33.3) | 666 (9.0) | 148 (5.6) |
|  | 2016/17 | 15 (20.3) | 0 (0.0) | 1020 (13.7) | 307 (11.6) |
|  | 2018/19 | 14 (18.9) | 1 (33.3) | 1639 (22.0) | 563 (21.2) |
|  | 2020/21 | 24 (32.4) | 1 (33.3) | 3456 (46.4) | 1463 (55.1) |
| Cocaine | All | 3681 (100.0) | 134 (100.0) | 4263 (100.0) | 682 (100.0) |
|  | 2012/13 | 303 (8.2) | 1 (0.8) | 295 (6.9) | 39 (5.7) |
|  | 2014/15 | 455 (12.4) | 17 (12.7) | 346 (8.1) | 68 (10.0) |
|  | 2016/17 | 670 (18.2) | 20 (14.9) | 784 (18.4) | 121 (17.7) |
|  | 2018/19 | 1072 (29.1) | 47 (35.1) | 1130 (26.5) | 180 (26.4) |
|  | 2020/21 | 1181 (32.1) | 49 (36.6) | 1627 (38.2) | 256 (37.5) |
| Gabapentin | All | 652 (100.0) | 13 (100.0) | 207 (100.0) | 7 (100.0) |
|  | 2012/13 | 34 (5.2) | 0 (0.0) | 2 (1.0) | 0 (0.0) |
|  | 2014/15 | 68 (10.4) | 0 (0.0) | 4 (1.9) | 0 (0.0) |
|  | 2016/17 | 116 (17.8) | 4 (30.8) | 36 (17.4) | 0 (0.0) |
|  | 2018/19 | 215 (33.0) | 6 (46.2) | 59 (28.5) | 2 (28.6) |
|  | 2020/21 | 219 (33.6) | 3 (23.1) | 103 (49.8) | 5 (71.4) |
| Any synthetic cannabinoid receptor agonist/s (SCRA) implicated | All | 224 (100.0) | 37 (100.0) | 32 (100.0) | 4 (100.0) |
|  | 2012/13 | 1 (0.5) | 0 (0.0) | 0 (0.0) | 0 (0.0) |
|  | 2014/15 | 15 (6.7) | 1 (2.7) | 0 (0.0) | 1 (25.0) |
|  | 2016/17 | 51 (22.8) | 13 (35.1) | 10 (31.3) | 1 (25.0) |
|  | 2018/19 | 77 (34.4) | 13 (35.1) | 14 (43.8) | 2 (50.0) |
|  | 2020/21 | 80 (35.7) | 10 (27.0) | 8 (25.0) | 0 (0.0) |
| Only synthetic cannabinoid receptor agonist/s (SCRA) implicated | All | 89.0 (100.0) | 18 (100.0) | 27 (100.0) | 2 (100.0) |
|  | 2012/13 | 1 (1.1) | 0 (0.0) | 0 (0.0) | 0 (0.0) |
|  | 2014/15 | 6 (6.7) | 0 (0.0) | 0 (0.0) | 1 (50.0) |
|  | 2016/17 | 5 (28.1) | 6 (33.3) | 10 (37.0) | 0 (0.0) |
|  | 2018/19 | 28 (31.5) | 7 (38.9) | 13 (48.2) | 1 (50.0) |
|  | 2020/21 | 29 (32.6) | 5 (27.8) | 4 (14.8) | 0 (0.0) |
| Any opioid/s + Any non-opioid substance/s implicated | All | 9326 (100.0) | 410 (100.0) | 5704 (100.0) | 1300 (100.0) |
|  | 2012/13 | 1371 (14.7) | 60 (14.6) | 161 (2.8) | 16 (1.2) |
|  | 2014/15 | 1654 (17.7) | 69 (16.8) | 263 (4.6) | 50 (3.9) |
|  | 2016/17 | 1761 (18.9) | 93 (22.7) | 737 (12.9) | 109 (8.4) |
|  | 2018/19 | 2190 (23.5) | 107 (26.1) | 1323 (23.2) | 209 (16.1) |
|  | 2020/21 | 2350 (25.2) | 81 (19.8) | 3078 (54.0) | 862 (66.3) |

**References**

1. Von Elm E, Altman DG, Egger M, Pocock SJ, Gøtzsche PC, Vandenbroucke JP. The Strengthening the Reporting of Observational Studies in Epidemiology (STROBE) statement: guidelines for reporting observational studies. Annals of internal medicine. 2007;147(8):573-7.
